# Ageing Trajectories: Exposome-Driven Pathobiological Mechanisms and Implications for Prevention from Blue Zones and Italian Longevity Hotspots Such as Cilento and Sicilian Mountain Villages

**DOI:** 10.3390/ijms26104796

**Published:** 2025-05-16

**Authors:** Silvana Mirella Aliberti, Mario Capunzo, Damiano Galimberti, Giulia Accardi, Anna Aiello, Anna Calabrò, Calogero Caruso, Giuseppina Candore

**Affiliations:** 1 Department of Medicine, Surgery and Dentistry “Scuola Medica Salernitana”, University of Salerno, 84081 Salerno, Italy; sialiberti@unisa.it (S.M.A.); mcapunzo@unisa.it (M.C.); 2 International Longevity Science Association, 20159 Milan, Italy; damiano.galimberti@gmail.com; 3 Laboratory of Immunopathology and Immunosenescence, Department of Biomedicine, Neurosciences and Advanced Diagnostics, University of Palermo, 90134 Palermo, Italy; giulia.accardi@unipa.it (G.A.); anna.aiello@unipa.it (A.A.); anna.calabro@unipa.it (A.C.); giuseppina.candore@unipa.it (G.C.)

**Keywords:** ageing, exposome, longevity, blue zones, Cilento, Sicani and Madonie Mountains, environmental pollution, non-communicable diseases, immune-inflammatory responses

## Abstract

Ageing is influenced by a complex interplay of genetic, environmental, and lifestyle factors, with increasing evidence highlighting the exposome as a key determinant of healthspan. This review explores the impact of environmental exposures, focusing in particular on pollution, endocrine disruptors, and climate change on the development of age-related diseases such as cardiovascular diseases (CVDs), cancer, and metabolic disorders. Additionally, it examines protective factors that contribute to healthy ageing. A comprehensive literature review was conducted using PubMed, Scopus, and Google Scholar, focusing on studies published between 2000 and 2025. Findings indicate that chronic exposure to pollutants accelerates cellular ageing through mechanisms such as oxidative stress, inflammation, and epigenetic dysregulation. In contrast, longevity hotspots—Blue Zones, Cilento and the mountain villages of Sicily (Sicani and Madonie Mountains)—illustrate how traditional dietary patterns, strong social structures, and reduced environmental toxicity contribute to exceptional health and longevity. Mechanistically, exposome-driven alterations in immune-inflammatory pathways and epigenetic regulation play a central role in modulating ageing trajectories. Understanding these interactions is essential for developing targeted strategies to mitigate harmful exposures and enhance protective factors. This review underscores the urgent need for integrative public health policies that address the environmental determinants of ageing, ultimately promoting a longer and healthier lifespan.

## 1. Introduction: Healthy and Unhealthy Ageing

The global landscape of ageing is experiencing a transformative shift. By 2050, the population aged 60 years and older is projected to surpass 2 billion, with 434 million people aged 80 years and above [1]. This unprecedented demographic transition presents both challenges and opportunities, urging societies, healthcare systems, and economies to rethink strategies for addressing the complexities and potential of an ageing world.

However, increased life expectancy does not equate to an extended healthy life expectancy. While a modest decline in severe disability rates has been observed in high-income countries over the past three decades, mild to moderate disabilities remain prevalent [2]. Epidemiological studies reveals that 15–35% of individuals aged 75 and older in the Western countries require assistance with daily activities, highlighting a gap between lifespan and quality of life [3]. This underscores the need to shift focus from lifespan to healthspan, which integrates the dimensions of physical, mental, and social well-being.

Ageing, an unavoidable natural phenomenon, stems from the disruption of the self-organisation system and reduced ability to adapt to the environment. As humans age, harmful changes accumulate in the molecules, cells, and tissues, responsible for declining normal physiological functions and reduced ability to maintain adequate homeostasis, resulting in greater susceptibility to different stressors. This decline is shaped by a dynamic interplay of genetic, epigenetic, stochastic, and environmental factors, leading to diverse ageing trajectories across individuals. Healthy ageing refers to maintaining functional ability that enables individuals to thrive in older age, while unhealthy ageing is characterised by frailty, disability, and chronic disease [4,5,6,7,8].

Thus, the multifaceted process of ageing is significantly influenced by environmental and lifestyle factors [8]. This leads to the consideration of the exposome [9], which represents the cumulative environmental exposures across an individual lifespan, interacting with genetic predispositions to shape health and ageing trajectories.

This narrative review examines how environmental exposures contribute to biological ageing and the development of age-related non-communicable diseases (NCDs), including cardiovascular disease (CVD), cancer, type 2 diabetes (T2D), and respiratory disorders. Rather than attempting to comprehensively map the entire exposome, the review focuses on high-impact, well-documented exposures, such as air and water pollution, xenobiotics and endocrine disruptors, microplastics, and climate change, as well as their mechanistic pathways, including oxidative stress, chronic inflammation, metabolic disruption, and epigenetic dysregulation. It also draws comparative insights from natural longevity models, including the Blue Zones, Cilento, and Sicilian villages in the Sicani and Madonie Mountains [10,11,12], which offer real-world examples of protective environmental and lifestyle patterns. By integrating evidence from environmental health, the biology of ageing, and preventive medicine, this review seeks to inform future public health strategies and policies through an exposome-informed lens.

## 2. Materials and Methods

This narrative review synthesises current evidence on selected environmental exposures relevant to ageing and longevity. However, it follows a structured and targeted search strategy to guide evidence selection and ensure consistency across the environmental, biomedical, and epidemiological domains.

### 2.1. Literature Search Strategy

A comprehensive search was performed using PubMed, Scopus, and Google Scholar, covering peer-reviewed articles published in English from 2000 to 2025. The search included the following thematic categories and keyword combinations:

- General framework: “exposome”, “ageing”, “healthy ageing”, “unhealthy ageing”, “environmental exposures”, “chronic diseases”, “NCDs”.
- Environmental risk factors: “pollution, “climate change”, “extreme heat”, “wildfires”, “air pollution”, endocrine disruptors”, “phthalates”, “bisphenol A”, “persistent organic pollutants”, “microplastics”.
- Biological mechanisms: “oxidative stress”, “inflammation”, “immune-inflammatory responses”, epigenetic dysregulation”, “metabolic regulation”.
- Disease outcomes: “cardiovascular diseases (CVDs)”, “cancer”, “type 2 diabetes (T2D)”, “chronic respiratory diseases”.
- Built environment: “urban design”, “green space”, “walkability”, “housing quality”, “transport infrastructure”, “noise pollution”, “access to healthcare”.
- Positive longevity models: “Blue Zones”, “Cilento”, “Sicani Mountains”, “Madonie Mountains”, “Mediterranean diet”, “healthy lifestyle”, “longevity hotspots”, “socio-environmental resilience”.

### 2.2. Study Selection Process

A multi-step process was used:

1. Identification: Titles and abstracts were screened for relevance to exposome-ageing interactions.
2. Screening: Duplicates were removed, and non-English or non-peer-reviewed studies were excluded.
3. Eligibility Assessment: Full-text articles were assessed based on methodological quality and thematic relevance.
4. Inclusion: The selected studies were incorporated into the thematic sections of the review.

#### 2.2.1. Inclusion Criteria

Studies were included if they

▪ Investigated environmental exposures in relation to healthy/unhealthy ageing or age-related diseases.
▪ Examined biological mechanisms such as oxidative stress, immune-inflammatory responses, metabolic and epigenetic regulation.
▪ Addressed high-impact exposures such as pollutants, endocrine disruptors, climate stressors, and built environment factors.
▪ Provided comparative insights into protective models of longevity (e.g., Blue Zones, Cilento and Sicilian Mountain villages).

#### 2.2.2. Exclusion Criteria

Studies were excluded if they

▪ Focused solely on genetics without considering environmental interactions.
▪ Lacked methodological transparency or relevance to age-related outcomes.
▪ Were limited to occupational or early-life exposures without adult or lifespan implications.

Two independent reviewers (SMA and CC) screened and selected the articles. Discrepancies were resolved through discussion. Additional reviews and book chapters were used to contextualise and supplement the findings.

## 3. The Exposome: A Comprehensive Framework

The exposome, a concept introduced by epidemiologist Christopher Wild in 2005 [9], encapsulates the sum of environmental influences from conception to ageing. This framework includes not only external and internal environmental factors but also the interaction of these factors with the genome, starting from the prenatal stage. In 2012, Wild [13] further delineated the exposome into three integral domains:

- The General External Environment, which comprises macro-level factors such as urban–rural dichotomy, climatic conditions, socioeconomic status, and educational attainment.
- The Specific External Environment, focusing on individual lifestyle factors including diet, physical activity, smoking (encompassing traditional, electronic, and tobacco-heating systems), occupational exposures, infections, and other personal behaviours.
- The Internal Environment, which pertains to internal biological processes like metabolic activity, gut microbiota composition, inflammatory responses, oxidative stress, and the ageing process itself.

A growing body of evidence highlights the built environment as a key component of the general external exposome, shaping patterns of physical activity, social engagement, exposure to pollutants, and access to green spaces and healthcare services. Elements such as urban design, walkability, housing quality, transportation infrastructure, and proximity to environmental pollution sources significantly influence long-term health trajectories and may contribute to disparities in ageing outcomes [14,15]. For instance, living in areas with limited green space and high traffic density has been associated with increased risks of cardiovascular disease, cognitive decline, and reduced healthspan in older adults. In contrast, age-friendly urban planning, characterised by safe pedestrian zones, clean air, and accessible parks, has been linked to improved physical and mental well-being in ageing populations [15]. The beneficial impact of green spaces on ageing and longevity is well documented; access to natural environments has been linked to slower epigenetic ageing and improved mental health, particularly in areas with greater forest cover [16,17]. Overall, the built environment serves as a structural determinant of health, operating through multiple pathways that intersect with biological, behavioural, and social factors throughout the life course.

Building on Wild’s foundational model, Miller and Jones [18] refined the exposome framework by integrating behavioural risk factors and endogenous metabolic responses, thereby linking external exposures with internal physiological processes. Their work highlights the bidirectional relationship between environment and biology, where exposures not only influence but are also shaped by host responses. This expanded perspective enables a more dynamic and integrative understanding of how diverse exposures, from urban air pollution to individual behaviours such as diet and smoking, interact to influence health outcomes across the ageing continuum.

Evidence from numerous studies highlights that genetic variations alone cannot fully explain the variability in the onset and progression of chronic diseases. Environmental exposures and their interactions with genetic factors play a crucial role. For instance, in the case of allergic diseases, while genetic studies have revealed certain molecular pathways, only a minor portion of the disease variability is explained by identified genetic markers [19].

However, the full spectrum of environmental contributors remains elusive due to challenges such as measurement errors, limitations in risk models, and the lack of tools to assess unrecognised exposures or the synergistic effects of environmental, biological, and behavioural factors [20].

Epidemiological research often focuses on individual agents, overlooking the impact of co-exposures and their cumulative effects over time. The exposome framework, by contrast, considers the dynamic nature of lifelong exposures, including fluctuations, peaks, and their interactions, offering a more comprehensive perspective on disease risk and prevention.

A significant component of the exposome involves xenobiotics, foreign substances to an organism, derived from Greek words meaning ‘foreign’ (ξένος—xenos), and ‘life’ (βίος—bios). These include pharmaceuticals and other compounds not naturally expected within the organism. Xenobiotics might affect one species adversely while being benign in another, as seen with human hormones affecting fish populations through wastewater [21].

Commonly, several xenobiotics are human-made pollutants with potential detrimental effects on biological systems. These include pharmaceuticals, pesticides, cosmetics, industrial by-products, food additives, and environmental contaminants. Throughout a lifetime, humans are estimated to encounter between 1 and 3 million xenobiotic substances, mainly via diet, air, water, medications, and lifestyle choices [22]. The body detoxification pathways play a crucial role in mitigating their toxicity and facilitating their elimination. However, individual variations in metabolism, genetic predisposition, and cumulative exposure levels influence the extent to which these substances impact health, reinforcing the need for an exposome-based approach to disease prevention and risk assessment.

The exposome framework provides a comprehensive perspective on how environmental exposures interact with biological mechanisms to influence health outcomes. By integrating multiple exposure sources and their cumulative effects over time, this approach enables a more holistic understanding of disease aetiology. It also underscores the potential for preventive strategies aimed at mitigating the adverse impact of environmental factors on human health. The influence of the exposome on ageing extends beyond cellular mechanisms, impacting broader physiological processes through its interplay with lifestyle and environmental exposures.

## 4. Exposome and Ageing: Insights from Research

The ageing process is characterised by a progressive decline in intrinsic biological functions, driven by mechanisms such as genomic instability, loss of proteostasis, telomere attrition, epigenetic alterations, and disabled macroautophagy, primary hallmarks of ageing [23]. They are significantly modulated by environmental and lifestyle factors, emphasising the central role of the exposome [24]. The exposome plays a pivotal role in ageing, either accelerating biological decline or enhancing resilience through adaptive mechanisms.

Research has demonstrated that ageing and age-related diseases can share common cellular and molecular pathways influenced by environmental factors [23,25]. Prolonged exposure to air pollutants like fine particulate matter (PM2.5), tobacco smoke, and toxic chemicals accelerates cellular senescence, genomic instability, and chronic inflammation. PM2.5, for instance, has been linked to millions of premature deaths annually, exacerbating CDVs, neurodegenerative conditions, and skin ageing [26].

Telomere attrition, a widely recognised hallmark of ageing, is particularly linked to environmental stressor [23]. Long-term exposure to pollutants, occupational hazards, and smoking accelerates telomere shortening, amplifying cellular senescence, a state in which cells permanently stop dividing but do not die. The accumulation of senescent cells over time contributes to ageing and age-related diseases by promoting inflammation and tissue dysfunctions [27]. Additionally, mitochondrial dysfunction, exacerbated by environmental pollutants, further disrupts cellular energy production and increases oxidative damage, thereby compounding ageing processes [28]. However, lifestyle and dietary can mitigate these effects. The Mediterranean diet, rich in polyphenols, omega-3 fatty acids, and antioxidants, has been associated with telomere preservation and reduced systemic inflammation, offering protection against environmental accelerators of ageing [29,30].

In this context, nutrigenomics and nutrigenetics provide powerful tools to understand how diet influences gene expression and individual responses to nutrients. Nutrigenomics explores how bioactive compounds in food modulate genes related to oxidative stress and inflammation, while nutrigenetics examines genetic polymorphisms that affect dietary responses. Together, these approaches enable personalised nutritional strategies to counteract the harmful effects of the exposome and promote healthy ageing [31].

The exposome systemic effects are not limited to cellular damage but extend to inflammatory dysregulation. Chronic environmental exposures can contribute to a persistent state of low-grade inflammation, termed “inflamm-ageing” [32]. This pro-inflammatory state, characterised by elevated levels of cytokines and chemokines, accelerates tissue degradation and heightens the risk of age-related diseases such as type 2 diabetes (T2D), CDV, and autoimmune conditions [33].

While environmental exposures pose significant challenges, targeted interventions can counteract their negative impacts. A combination of environmental protection policies, optimised nutrition, and lifestyle modifications offers a comprehensive strategy to mitigate the effects of the exposome and support healthier ageing trajectories on a global scale.

## 5. The Exposome and Pollution: A Pervasive Interaction

Building on the broader understanding of how the exposome influences ageing, pollution emerges as one of the most pervasive environmental factors shaping ageing trajectories through its effects on cellular health, systemic resilience, and lifespan. Environmental exposures, particularly pollution, may act as a key determinant in differentiating healthy ageing from unhealthy ageing [34]. Air pollution, one of the most studied forms of environmental contamination, has been consistently linked to increased risk of NCDs, cognitive decline, frailty, and all-cause mortality, which are hallmarks of unhealthy ageing. For instance, individuals aged ≥75 years have shown heightened susceptibility to mortality related to both short- and long-term exposure to PM2.5 compared to younger populations, emphasising the vulnerability of older adults to environmental stressors [35]. Chronic exposure to fine particulate matter (PM2.5 and PM10) further reinforces this susceptibility. Among the key contributors to air pollution are nitrogen oxides (NOx) and sulphur oxides (SOx), major pollutants stemming from traffic, combustion of fossil fuels, and industrial emissions. These compounds play a significant role in the formation of fine particulate matter (PM2.5) and acid rain, exacerbating respiratory and cardiovascular health issues, which are particularly detrimental to older people [36].

However, methodological limitations, such as confounding factors like socioeconomic disparities, publication bias, and inadequate exposure assessment techniques, challenge the establishment of causal relationships. Future research should leverage advanced metrics, such as frailty indices and temporal activity patterns, to better capture the multifaceted impacts of pollution on ageing. Expanding investigations to include low- and middle-income countries is crucial, as these regions often experience disproportionate pollution burdens with limited data availability.

A landmark U.S. study analysing nearly 28 million individuals aged ≥55 years across 3034 counties revealed that higher PM2.5 levels were inversely associated with the likelihood of living to age 85 or beyond, even after adjusting for socioeconomic, behavioural, and geographic variables. Strikingly, this inverse association was observed even at PM2.5 concentrations below the regulatory limits set by agencies such as the U.S. Environmental Protection Agency and the European Union, suggesting that even low levels of pollution can undermine health outcomes. The study also highlighted the compounding effects of other factors like poverty, smoking, and obesity, underscoring the need for a multifaceted approach to address both pollution and social determinants of health to promote exceptional lifespan [37]. While these studies did not record specific causes of death, the adverse effects of pollution on immune-inflammatory responses likely contribute to increased mortality from NCDs and infectious diseases in older populations [34,35].

In addition to airborne pollutants, water pollution represents another critical dimension of the exposome with profound implications for ageing and cardiovascular health. Contaminants such as arsenic, lead, cadmium, mercury [38,39,40,41,42,43,44,45,46,47,48,49,50,51,52,53,54,55,56], and organic pollutants like polycyclic aromatic hydrocarbons (PAHs), pesticides, and disinfection by-products (DBPs) are known to generate excessive reactive oxygen species (ROS), leading to oxidative stress, systemic inflammation, and endothelial dysfunction [57,58,59]. In particular, exposure to DBPs, such as trihalomethanes (THMs) and haloacetic acids (HAAs), amplifies the systemic impact of environmental pollutants [60,61].

Chronic exposure to these toxicants may impair the nuclear factor erythroid 2-related factor 2 (Nrf2) pathway, which plays a key role in antioxidant defence and cellular resilience due to its ability to activate downstream antioxidant enzymes, such as superoxide dismutase, catalase, and glutathione peroxidase, which are essential for maintaining vascular homeostasis [62,63,64]. Dysregulation of Nrf2 is particularly relevant in older people, where the natural decline of antioxidant pathways exacerbates susceptibility to pollutant-induced cardiovascular damage. For instance, diminished adaptive antioxidant responses in aged vasculature facilitate oxidative damage accumulation, contributing to endothelial senescence, vascular inflammation, and atherosclerosis [65,66,67,68,69].

Microplastics, a pervasive class of environmental pollutants, extend beyond water contamination to include inhalation as a major route of exposure, since they are present in indoor and outdoor air due to sources such as tire wear, synthetic textiles, and industrial emissions [70]. Unlike their role as mere carriers of toxic substances, microplastics exhibit intrinsic cytotoxicity, triggering apoptosis, mitochondrial dysfunction, and DNA damage. Recent research has highlighted their ability to penetrate biological barriers and accumulate in vital organs, exacerbating respiratory and systemic inflammation. Additionally, experimental studies suggest that microplastics may interfere with redox homeostasis, disrupting the Nrf2 pathway. Chronic inhalation exposure has been linked to respiratory disorders such as lung cancer, asthma, and hypersensitivity pneumonitis, linked to oxidative stress and inflammation [71,72]. Their interaction with oxidative stress pathways underscores the need for further research into their role in ageing and cardiovascular health. Given the widespread presence of microplastics in human tissues and their growing implications for chronic disease, refining exposure assessment methods and exploring mitigation strategies are crucial.

Xenobiotics, including industrial chemicals, hydrocarbons, and pharmaceuticals, represent another critical dimension of pollution, significantly influencing ageing and health outcomes. Many xenobiotics act as endocrine-disrupting chemicals (EDCs), interfering with hormonal homeostasis [73] and contributing to developmental abnormalities and carcinogenesis. Ageing exacerbates susceptibility to xenobiotics due to the diminished detoxification capacity [74,75]. Xenobiotic-metabolising enzymes (XMEs) play a vital role in mitigating the toxic effects of these compounds, but their efficiency is influenced by genetic variability, which can alter an individual’s ability to process and eliminate harmful substances. A recent cohort study [76] of 1112 individuals aged 20–108 years identified specific genetic variants in XME genes that accounted for 7.7% of the likelihood of surviving past 89 years. These findings highlight the complex interplay between genetics, environmental exposures, and ageing.

The prevailing view suggests that pollution-related health effects arise through an inflammation-driven cascade and oxidative stress affecting lung, vascular, and cardiac tissues. Inflammation initially serves as a protective response, eliminating harmful stimuli while generating ROS that can trigger cell death. During the early inflammatory phase, oxidative stress does not directly harm cells but instead activates stress defence genes, including those encoding antioxidants. This ROS-mediated preconditioning bolsters resilience against future oxidative stress and facilitates tissue repair. However, the subsequent release of cellular contents exacerbates inflammation, potentially leading to tissue damage [77].

The pivotal role of the Nrf2 pathway in mitigating oxidative stress highlights potential therapeutic strategies. Dietary bioactive compounds, such as sulforaphane (found in cruciferous vegetables) and curcumin (from turmeric), have demonstrated efficacy in activating Nrf2, enhancing antioxidant defences, and protecting against pollutant-induced cardiovascular damage [78,79]. These interventions could complement broader public health measures aimed at reducing pollution and addressing its impact on vulnerable populations.

Finally, a 2016 study investigated the relationship between epigenetic ageing and air pollution exposure in men, finding a strong link between accelerated ageing and PM2.5 [80]. Using newly developed DNA methylation-based ageing measures, the study provided novel evidence linking air pollution to DNA methylation. Notably, increased PM10 and NO2 levels were associated with accelerated epigenetic ageing exclusively in black women, with no such effect observed in non-Hispanic white women. These findings underscore the critical link between environmental exposure and social inequities, reinforcing the urgent need for environmental justice initiatives to reduce harmful exposures disproportionately affecting marginalised communities [37,81].

In conclusion, pollution, encompassing air, water, and xenobiotics, emerges as a significant determinant of ageing, influencing molecular and cellular processes, systemic resilience, and lifespan. Addressing these challenges requires a multi-pronged approach combining public health policies, environmental regulations, and personalised interventions targeting pathways like Nrf2. Such efforts hold the potential to mitigate the adverse effects of pollution, promote cardiovascular health, and enhance the likelihood of achieving healthy ageing.

## 6. Climate Change as a Component of the Exposome

Climate change emerges as a critical and inseparable component of the exposome, amplifying the health challenges posed by environmental pollutants and contributing to inflammatory responses. The intricate relationship between rising global temperatures, shifting weather patterns, and increasing pollution levels emphasises climate changes as both a driver and consequence of exposome. These effects extend beyond acute impacts, embedding themselves into broader systems that shape health outcomes across the lifespan [82].

Climate change exerts profound and multifaceted impacts on human health, directly through extreme weather events and indirectly via disruptions to ecosystems, economies, and social systems. Global temperatures have already risen by 1.1 °C compared to pre-industrial levels, with projections indicating further increases between 2.5 °C and 2.9 °C by the end of the century if greenhouse gas emissions are not significantly reduced. The summer of 2024, the hottest recorded in the Northern Hemisphere since 1850, and likely the hottest in the past 2000 years [83,84], illustrates the acute effects of global warming. Events like these reflect trends driven by human activities, particularly the combustion of fossil fuels, which the Intergovernmental Panel on Climate Change has unequivocally identified as the primary driver of global warming [84,85,86].

Extreme heat events, such as those recorded in recent years, have far-reaching implications for health [85]. Heatwaves lead to a spectrum of heat-related diseases, including heat stroke, heat exhaustion, heat cramps, and heat rash. More insidiously, heat exacerbates pre-existing conditions, such as cardiovascular and respiratory diseases, contributing to increased morbidity and mortality. Vulnerable populations, including children, older adults, and those with chronic diseases, are disproportionately affected. Even young, healthy individuals can succumb to extreme heat when temperatures remain elevated for prolonged periods without nocturnal cooling. For example, the 2003 European heatwave caused over 14,000 deaths in France alone, underscoring the lethality of heat in temperate regions unprepared for such extremes [84,87].

The physiological impacts of heat extend beyond acute diseases, driven by mechanisms such as oxidative stress and systemic inflammation [88]. Research shows [89,90] that even temperatures as low as 34 °C in humid conditions can elevate cardiovascular stress before the core body temperature rises. This stress, induced by increased heart rates and blood flow redistribution to regulate temperature, can become fatal for individuals with underlying heart conditions. For example, Cottle et al. [89] conducted a study where 51 healthy young participants performed light physical activity in an environmental chamber with temperature and humidity gradually increasing. They found that heart rate increased at 34 °C under humid conditions, compared to 41 °C in dry air, highlighting the amplified cardiovascular strain at lower temperatures due to humidity. Henderson et al. [90] further showed that heat affects the heart even at rest; at 50 °C with 50% humidity, the resting heart rate was 64% higher than at 28 °C. While the body’s natural response to regulate heat—such as sweating and increased blood flow—is manageable for healthy young individuals, it can be life-threatening for older adults or those with heart conditions. The rising frequency of heat domes, which trap warm air and worsen heatwaves, intensifies these risks, underscoring the urgent need for adaptative strategies and preventive measures.

Older adults are particularly vulnerable during heatwaves due to diminished thirst response and impaired renal function. These factors, combined with their reduced total body water and intravascular volume, make it harder for them to mitigate fluid losses through sweating. As a result, they face increased risk of dehydration, which is further exacerbated during extreme heat events [91,92].

A recent study indicates that prolonged exposure to intense heat may speed up biological ageing in older individuals, raising fresh concerns about the impact of climate change and heat waves on long-term health and ageing at a molecular scale. Residents of areas experiencing frequent high temperatures tend to exhibit greater biological ageing on average compared to those living in cooler regions. Biological age reflects body functionality at the molecular, cellular, and systemic levels, rather than simply the number of years since birth, i.e., chronological age. A biological age exceeding chronological age is linked to an elevated risk of disease and early mortality. Although extreme heat has long been recognised as a factor contributing to adverse health effects, including increased mortality rates, its connection to biological ageing has remained uncertain. These findings shed light on the biological mechanisms that may link heat exposure to ageing-related health risks and mortality [93].

As climate change accelerates, rising temperatures and more frequent heatwaves are placing urban populations at elevated risk. Cities, characterised by heat-absorbing materials such as concrete and asphalt, tend to be significantly warmer than surrounding rural or suburban areas. This urban heat amplification intensifies the threat of heat-related health conditions. In response, professionals from urban planning, climate science, meteorology, and public health are working to identify the communities most vulnerable to extreme heat. A key concern in this effort is the unequal impact of high temperatures, particularly on low-income populations and, in the U.S. context, African American communities. Decades of discriminatory zoning and infrastructure policies have resulted in these communities experiencing greater exposure to heat-related risks and higher rates of heat-related mortality than their white counterparts. Recent research has increasingly illuminated the structural inequities that leave some neighbourhoods dominated by heat-retaining surfaces and lacking in green infrastructure, while others benefit from extensive vegetation and recreational green spaces. Although such disparities are observed worldwide, the United States offers some of the clearest and best-documented examples of how historical discrimination translates into environmental vulnerability. In response, numerous cities have initiated climate adaptation efforts aimed at addressing thermal inequity, such as urban greening projects and reflective roofing in underserved areas. However, these interventions have yet to fully counteract the cumulative effects of decades of environmental neglect [82,94].

One of the most comprehensive investigations into urban heat disparities in the U.S. was conducted by Hsu et al. [95], who integrated remote sensing data on urban surface temperatures with census-based demographic information from 175 cities. Their findings revealed that in 97% of these cities, African American populations were exposed to average temperatures roughly one degree Celsius higher than those experienced by predominantly white, non-Hispanic communities. Furthermore, socioeconomic status played a significant role: individuals living below the poverty line, regardless of racial or ethnic identity, were subjected to higher temperatures compared to wealthier residents. This temperature gap is attributed to several factors, including a scarcity of tree cover, extensive impervious surfaces, and planning decisions that have concentrated highways and industrial zones, both significant heat sources, in areas housing communities of colour [94,95].

These environmental and infrastructural inequalities heighten the vulnerability of marginalised groups to heat-related health problems. Additional contributing factors include limited healthcare access, a higher likelihood of working in heat-exposed occupations (such as outdoor labour or poorly ventilated industrial facilities), and increased prevalence of chronic conditions such as diabetes, hypertension, and renal disease [94,96].

Beyond direct heat-related diseases, climate change indirectly affects health by disrupting food security, water availability, and air quality. Rising temperatures and shifting rainfall patterns threaten agricultural systems, leading to reduced crop yields and food shortages, which in turn contribute to malnutrition and micronutrient deficiencies [97]. Furthermore, water scarcity increases the prevalence of waterborne diseases such as cholera and dysentery [98]. Air quality degradation is another critical pathway through which climate change undermines health, as wildfires and stagnant air masses release pollutants that exacerbate respiratory and cardiovascular conditions [99]. These interconnected disruptions amplify heath vulnerabilities, particularly in low-resource settings where adaptive capacity is limited [82].

Climate change also facilitates the spread of infectious diseases by altering ecosystems and expanding the habitats of disease-carrying vectors. Warmer temperatures and changing precipitation patterns enable mosquitoes to thrive in previously inhospitable regions, increasing the transmission of diseases such as malaria, dengue fever, and Zika virus. Similarly, tick populations, which carry Lyme disease, are expanding their geographic range, posing new challenges for public health systems [100,101]. These changes highlight the interconnectedness of climate, ecosystems, and human health within the exposome framework.

On a broader scale, climate change contributes to the global burden of NCDs by exacerbating inflammation-related pathways linked to CDV, cancer, T2D, and respiratory diseases [82,102,103,104,105,106,107,108].

The interplay between the exposome, inflammation, and NCDs underscores the urgent need for integrated strategies to mitigate these risks (Table 1).

## 7. Exposome and Immuno-Inflammatory Responses

In the previous sections, we examined the health effects of pollution, microplastics, xenobiotics, and climate change on older adults, emphasising how many of these impacts are driven by oxidative stress and a pro-inflammatory state known as inflamm-ageing. Adults aged 75 and older are particularly vulnerable to pollution-related frailty, cognitive decline, and increased mortality, highlighting the need for more precise exposure assessments and the inclusion of frailty indices in future research [102,109].

Some studies have also assessed socioeconomic status (SES) and found that it exacerbates the negative effects of these components of exposome. In this regard, a recent multicohort study explored the links between social disadvantage, age-related plasma proteins, and age-associated diseases. The findings identified proteomic signatures indicative of accelerated immune ageing, with 14 specific age-related proteins associated with social disadvantage in both early and later life. Individuals experiencing social disadvantage faced an elevated risk for 66 age-related diseases, with up to 39% of these associations mediated by the 14 identified proteins. The primary enriched pathway revealed the upregulation of the pro-inflammatory regulator NF-κB24 and its downstream effector, interleukin-8 [110].

This study corroborates and expands on previous research demonstrating that lower socioeconomic status and lower education levels are linked to increased baseline inflammation. Several mechanisms may explain the influence of social environment on inflammation. Socioeconomically disadvantaged individuals are disproportionately exposed to pro-inflammatory environmental factors, including pollution, xenobiotics, climate change, and infectious agents due to overcrowded living conditions, poor housing quality, and inadequate sanitation. Additionally, unhealthy behaviours, more prevalent in lower SES groups, further increase exposure to pro-inflammatory and oxidative stressors. These include smoking and dietary patterns that contribute to visceral obesity, a well-known driver of chronic inflammation. Chronic social disadvantage can also lead to prolonged psychological stress, triggering an amplified stress response and further elevating baseline inflammation [111,112,113,114,115,116].

Regarding the mechanisms underlying the pro-inflammatory state induced by pollution, a recent study used RNA-seq to characterise the transcriptional response of peripheral blood mononuclear cells exposed to PM10, identifying 1196 differentially expressed genes. The findings suggest that PM10 exposure triggers an inflammatory response, marked by the upregulation of cytokines, chemokines, and, notably, IL-36 subfamily members (IL36B, IL36G, and IL36RN), while downregulating genes critical for pathogen defence, including antimicrobial peptides (AMPs) and antiviral factors. In vitro experiments with respiratory epithelial cells corroborated these findings, demonstrating that PM10 exposure increases the production of inflammatory mediators while suppressing AMP expression. This dual effect—heightened inflammation alongside weakened antimicrobial defences—may contribute to the increased susceptibility to respiratory infections (e.g., influenza, RSV, and SARS-CoV-2) observed with prolonged PM10 exposure. Additionally, the study highlighted that PM10-induced inflammation is driven by key biological processes, including innate immune activation and inflammatory responses, with oxidative stress playing a central role [117].

In older people, this pro-inflammatory status (called inflamm-ageing) has a significant impact on multiple body systems, contributing to the onset and progression of numerous age-related diseases and the general decline of health. In addition, the inflammatory process is favoured by the decline of the immune system called immunosenescence and, in turn, aggravates this immune ageing, thus establishing a vicious cycle [27,118].

A well-functioning immune system is vital for a healthy body. Inadequate or excessive immune responses underlie diverse pathologies such as serious infections, cancer, and autoimmune conditions. Thus, it is crucial to understand how environmental pollutants impact the immune system to unravel the mechanisms by which pollution leads to disease and to identify potential strategies to mitigate these effects.

The immune system consists of diverse cell types that collaborate to generate (or fail to generate) effective immune responses. A recent study reviewed the potential impact of pollutants on immune cells. Specifically, air pollution exposure can amplify T helper type 2 (Th2) and Th17 responses while impairing antiviral defence. Th2 cells regulate humoral immunity by promoting B cell antibody class switching, particularly enhancing IgE production, whereas Th17 cells drive inflammation and play a key role in fighting extracellular bacteria and fungi by recruiting neutrophils. The clinical effects of air pollution, most notably its association with worsening asthma and chronic obstructive pulmonary disease, align with these immune changes. Additionally, as highlighted in the study, air pollution influences a broader range of immune functions, including neonatal immune development and gastrointestinal immunity [119].

Climate change worsens this problem by intensifying airborne allergens and pollution through more frequent and severe wildfires, dust storms, thunderstorms, and heatwaves. These environmental changes disrupt immune regulation, contributing to the increasing prevalence of immune-mediated diseases such as asthma, allergies, and autoimmune disorders [120].

Recent findings further illuminate the mechanisms of pollution-induced immune impairment. A study analysing lymph nodes (LNs) from 84 organ donors aged 11 to 93 years identified a distinct age-related decline in immune function specifically in lung-associated LNs, but not in those linked to the gut. This decline was attributed to the accumulation of inhaled atmospheric particulate matter. With advancing age, particulate density increased in lung-associated LNs, whereas gut-associated LNs remained unaffected. The particulates were exclusively found within macrophages, which exhibited diminished activation, impaired phagocytic capacity, and altered cytokine production, compared to macrophages without particulate accumulation. Additionally, lung-associated LNs with particulates displayed disrupted B cell follicle structures and impaired lymphatic drainage. These findings suggest that lifelong environmental exposure may progressively weaken lung immune surveillance by directly affecting immune cell function and lymphoid tissue architecture [121].

In summary, air pollution disrupts immune function through chronic inflammation, impaired immune responses, and heightened susceptibility to both autoimmune and infectious diseases. With global warming intensifying environmental stressors, addressing pollution and its immunological consequences remains a critical public health priority. Implementing stricter air quality regulations, mitigating climate change, and improving resilience against environmental hazards are essential strategies for protecting immune health across the lifespan.

## 8. Exposome and NCDs: The Rising Impact of Environmental Exposures on Ageing

The increasing prevalence of age-related NCDs presents a profound challenge to global health systems, particularly in ageing societies. In the USA alone, over 90% of older individuals suffer from at least one chronic NCD, and approximately three-quarters experience two or more [122]. This growing prevalence imposes a staggering economic burden, with costs associated with age-related NCDs estimated at $47 trillion between 2010 and 2030. These expenses encompass direct medical care, long-term disability, and productivity losses, placing immense strain on pension systems, social support structures, and healthcare systems globally [123].

Recognising the magnitude of this challenge, the World Health Organization (WHO) has emphasised the importance of transitioning from reactive care to proactive prevention, advocating for strategies that promote healthy ageing and reduce the burden of NCDs [124,125].

### 8.1. Exposome-Related Pathways in Chronic Diseases

The exposome framework provides a comprehensive lens through which to examine the biological underpinnings of ageing and age-related chronic diseases. Ageing is an inherently heterogeneous process that varies both between individuals and across organ systems within the same individual. While some older adults maintain high levels of physical and cognitive function, others experience a rapid decline into frailty and disease, despite being of the same chronological age [126,127].

Chronic NCDs account for 74% of the global disease burden annually, affecting millions of people worldwide. These conditions arise from a combination of behavioural factors (e.g., tobacco use, unhealthy diet, harmful alcohol consumption, and physical inactivity), biological factors (e.g., elevated blood pressure, obesity, high blood glucose, and cholesterol), and environmental factors (e.g., pollution, climate change and social disadvantage). The four leading causes of global mortality, CVD, cancers, T2D, and respiratory disease, have now surpassed infectious diseases as the predominant health threat [128]. Concerning tobacco use, alcohol consumption, nutrition, exercise, and sleep, these habits are acknowledged as key influences on NCDs associated with ageing. Researchers have started investigating the ways in which these lifestyle elements affect the progression of biological ageing. Diet and smoking were major contributors to overall negative associations of five lifestyle factors with biological age [126]. The rate of biological ageing differs across various organs within a single person, yet the degree to which accelerated ageing in particular organs heighten the likelihood of age-associated diseases in both the same and different organs is not well understood. A recent investigation reveals that advanced proteomic ageing of organs correlates with long-term vulnerability to age-related conditions. In many instances, the rapid ageing of one organ elevates the risk of diseases impacting multiple organ systems [129].

This complexity underscores the need to move beyond reductionist models based on single risk factors, and toward integrative approaches that consider cumulative, time-dependent exposures and their interactions with individual susceptibilities. The exposome framework, by integrating environmental, behavioural, and biological domains, can help uncover the molecular signatures and mechanistic pathways through which chronic age-related diseases emerge. This approach paves the way for more precise, prevention-oriented strategies that are tailored to individual risk profiles.

Advances in geroscience have further deepened our understanding of NCDs by identifying shared cellular and molecular mechanisms between ageing and chronic disease development. These include oxidative stress, mitochondrial dysfunction, impaired DNA repair, inflamm-ageing, cellular senescence, and epigenetic dysregulation [130]. Such hallmarks not only drive physiological ageing but also exacerbate the risk, onset, and progression of NCDs. Moreover, the integration of exposomic data with omics technologies has enabled the identification of molecular fingerprints linked to specific environmental exposures, thereby strengthening causal inference and highlighting potential preventive targets. For example, air pollution has been associated with distinct metabolomic and epigenetic signatures that mediate increased cardiopulmonary risk [131]. Likewise, endocrine-disrupting chemicals have been shown to interfere with insulin signalling, adipogenesis, and hepatic lipid metabolism, mechanisms contributing to the pathogenesis of obesity and T2D [132]. Together, these insights reveal how lifelong environmental exposures interact with intrinsic biological processes to shape the ageing trajectory and chronic disease risk, reinforcing the importance of exposome-informed prevention strategies.

### 8.2. Environmental Toxicants as Drivers of Major NCDs

Building upon the molecular insights provided by exposomic studies, this section examines specific classes of environmental toxicants that act as upstream drivers of the major NCDs. Climate change increasingly acts as a global amplifier of environmental exposures, such as air pollution, heatwaves, and water contaminants, modifying their distribution, intensity, and health impact, especially in ageing populations. As extensively discussed in the context of CVDs, the exposome framework provides a comprehensive lens through which the cumulative and interacting effects of lifelong environmental exposures can be mechanistically linked to chronic disease risk, particularly via oxidative stress, systemic inflammation, endothelial dysfunction, epigenetic modifications, and metabolic dysregulation [133,134,135].

These molecular mechanisms, which are critically implicated in the pathogenesis of CVDs, are increasingly recognised as common biological denominators across the full spectrum of NCDs.

Environmental exposures play a critical role in exacerbating the ageing mechanisms and promoting the development of chronic diseases. As previously described, airborne pollutants, PM2.5, NO2, and PAHs, contribute not only to cardiovascular morbidity and mortality but also to respiratory and metabolic dysfunctions. In the cardiovascular system, for example, PM2.5 has been shown to increase arterial stiffness and atherosclerotic burden via upregulation of inflammatory cytokines, disruption of mitochondrial function, and DNA methylation changes in genes regulating vascular integrity and immune homeostasis [133,134]. These effects, which have been characterised through high-resolution omics and longitudinal analyses, exemplify the systemic impact of environmental insults, particularly in ageing populations.

The integration of untargeted metabolomics and proteomics has further advanced our understanding of how specific pollutant classes, such as air pollution and traffic-related emissions, elicit distinct molecular signatures that correspond to subclinical markers of cardiovascular ageing, including coronary artery calcification and carotid intima-media thickness. Importantly, these signatures are not limited to CVDs but may serve as early biomarkers for broader environmental disease vulnerability [135,136]. Beyond cardiovascular, metabolic, respiratory, and cancer-related outcomes, growing evidence links environmental exposures to neurodegenerative diseases such as Alzheimer’s and Parkinson’s, musculoskeletal conditions including osteoporosis and sarcopenia, and chronic kidney disease. These disorders share common pathogenic pathways, including oxidative stress, mitochondrial dysfunction, endocrine disruption, and chronic inflammation, highlighting the broader relevance of the exposome in ageing biology. Phthalates, a prevalent group of EDCs found in plastics, have been shown to interfere with hormonal regulation and contribute to metabolic disorders, CVDs, T2D, and certain cancers. Similarly, other EDCs, such as bisphenol A (BPA), have been implicated in both cardiovascular dysfunction and metabolic alterations underlying T2D [132].

Recent findings have underscored the significance of waterborne pollutants, such as DBPs and heavy metals, in the aetiology of NCDs. Chronic arsenic exposure has been associated with an increased risk of cardiovascular and metabolic diseases, while mercury and cadmium have been shown to disrupt mitochondrial function, leading to exacerbated oxidative stress and inflammation [26,137]. The presence of microplastics, an increasingly recognised contaminant, has been demonstrated to act as endocrine disruptors and to serve as carriers for other harmful substances, thereby compounding their impact on human health [138].

Among the leading causes of global mortality, CVDs, cancer, T2D, and chronic respiratory diseases are profoundly influenced by environmental factors.

- Cardiovascular Diseases:
  CVDs, including ischemic heart disease and stroke, remain the leading cause of global mortality. Lifelong exposure to environmental toxicants plays a significant role in the development and progression of CVDs through a network of inter-related biological mechanisms. Among these, PM2.5, NO2, and phthalates have emerged as key contributors to CVD pathogenesis. Chronic exposure to air pollution accelerates atherosclerosis, endothelial dysfunction, and hypertension, primarily via increased oxidative stress and systemic inflammation. These toxicants also induce epigenetic alterations and metabolic dysregulation that impair vascular homeostasis and interact with age-related physiological changes, thereby compounding cardiovascular risk in older adults [133,134]. Furthermore, the identification of metabolomic and proteomic signatures associated with pollutant exposure and subclinical vascular ageing underscores the potential of exposome-informed approaches for early detection and personalised prevention of cardiovascular disease [139]. The role of phthalates in disrupting lipid metabolism and promoting arterial stiffness further underscores the importance of addressing environmental toxicants in CVD prevention [26].

- Cancer:
  Environmental carcinogens, including heavy metals (e.g., cadmium), metalloids (e.g., arsenic), microplastics, and persistent organic pollutants, play a significant role in cancer incidence and progression. These toxicants trigger DNA damage, disrupt cellular signalling pathways, and promote tumorigenesis. Hormone-dependent cancer, such as breast and prostate cancer, are particularly affected by endocrine disruptors like phthalates. The WHO estimates that environmental factors account for approximately 19% of all cancers [26,138,140]. However, the inflammatory effect of pollutants also plays a role in cancer, as inflammation is considered fuel for cancer [141].

- Diabetes:
  The global rise in T2D is closely linked to both behavioural and environmental factors. Chronic exposure to EDCs, such as phthalates and BPA, has been associated with insulin resistance, impaired glucose metabolism, and the development of T2D. These compounds disrupt hormonal signalling, alter adipose tissue function, and contribute to systemic inflammation. In older populations, T2D further increases the risk of other NCDs, including cardiovascular and renal complications, amplifying the overall disease burden [142].

- Chronic Respiratory Diseases:
  Conditions such as chronic obstructive pulmonary disease and asthma are exacerbated by long-term exposure to pollutants, including tobacco smoke, indoor air contaminants, and industrial emissions. These exposures increase the risk of airway inflammation, fibrosis, and decreased lung function, particularly in older adults, whose respiratory resilience is already diminished due to ageing [143].

### 8.3. The Exposome and Its Implications for Public Health: Strategies for Addressing NCDs in Older Populations

The concept of the exposome underscores the cumulative impact of lifetime exposures to environmental risks, lifestyle factors, and biological ageing processes. This holistic perspective highlights the need for integrative strategies to mitigate the burden of NCDs, particularly in older populations. In this context, climate change further complicates the exposome by amplifying exposure to heat stress, air pollution, and vector-borne diseases, disproportionately affecting older individuals and vulnerable communities [82].

Given the complex interplay between ageing, environmental exposures, and NCDs, a multi-pronged approach is essential to mitigate their impact. Strategies must include the following:

1. Primary Prevention: Reducing exposure to environmental toxicants through stronger regulations, improved air and water quality standards, and the promotion of safer consumer products.
2. Public Health Campaigns: Raising awareness of modifiable risk factors, such as smoking cessation, healthy diets, and increased physical activity.
3. Healthcare Innovation: Investing in early detection technologies, personalised medicine, and interventions that target the cellular mechanisms of ageing, such as mitochondrial therapies and antioxidants.
4. Global Equity: Addressing disparities in environmental exposures and access to healthcare, particularly in low- and middle-income countries that are disproportionately affected by pollution-related NCDs.

The increasing prevalence of NCDs in older people poses a critical challenge to global health systems and underscores the urgency of addressing environmental risk factors. The integration of geroscience, exposomics, and public health perspectives is essential to reduce the chronic disease burden, promote resilience in older individuals, and ensure equitable health outcomes. By systematically addressing environmental risk factors and advancing preventive strategies, global health efforts will be better positioned to achieve sustainable health and well-being for ageing populations in the 21st century.

**Figure 1:**
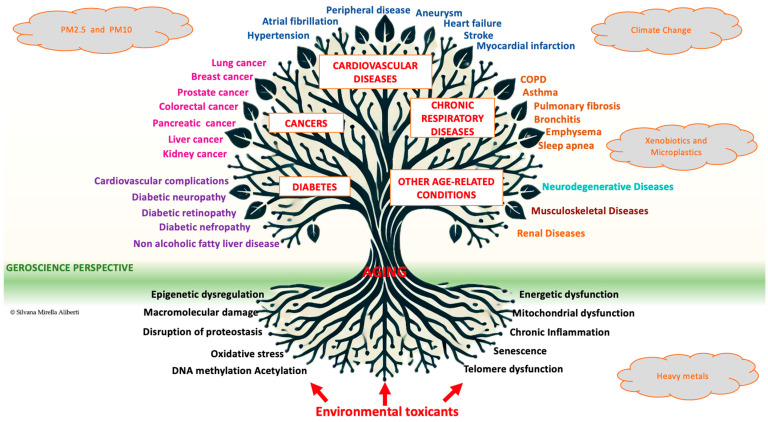
Conceptual model illustrating the role of environmental factors in unhealthy ageing. The figure presents a comprehensive framework linking environmental toxicants to unhealthy ageing and age-related diseases through their impact on fundamental molecular and cellular mechanisms.

Figure 1 depicts a conceptual model illustrating the role of environmental factors in unhealthy ageing.

The model is represented as a tree with the following elements:

1. Roots (Fundamental Ageing Mechanisms):

The roots symbolise core biological processes underlying ageing, exacerbated by environmental toxicants such as particulate matter, xenobiotics, and microplastics. These processes include oxidative stress, mitochondrial dysfunction, cellular senescence, epigenetic dysregulation, chronic inflammation, DNA methylation, acetylation, macromolecular damage, disruption of proteostasis, and energetic dysfunction.

2. Trunk (Connection to Systemic Diseases):

The trunk represents the transition from cellular ageing mechanisms to the development of systemic pathologies, highlighting the role of accelerated ageing in increasing vulnerability to chronic diseases.

3. Branches (Major Non-Communicable Diseases):

The branches extend upward, representing the four major global causes of mortality among NCDs:

  ○ CVDs (red label);
  ○ Cancer (red label);
  ○ T2D (red label);
  ○ Chronic respiratory diseases (red label);
  ○ Other age-related conditions (red label), including
    ○ Neurodegenerative diseases (turquoise text);
    ○ Musculoskeletal diseases (dark brown text);
    ○ Renal diseases (orange test).

4. Leaves (Specific Pathologies and Clinical Manifestations):
  The leaves represent organ-specific diseases and clinical outcomes associated with each category:
  ○ CVDs (blue text);
  ○ Cancer (fuchsia text);
  ○ T2D (purple text);
  ○ Chronic respiratory diseases (brown text).

5. Environmental Factors and Climate Change (External Drivers):
  Surrounding the tree, external drivers such as environmental toxicants and climate change are depicted in orange clouds, emphasising their synergistic and amplifying role across all levels of the model. These include fine particulate matter (PM2.5 and PM10), heavy metals, xenobiotics, microplastics, and climate change, which acts as a systematic stressor that exacerbates environmental risk, particularly for ageing populations.

6. Geroscience Perspective (Integrative Approach):
  Positioned beneath and around the tree, the geroscience perspective integrates biogerontology, environmental toxicology, and public health to address the complex interplay between environmental exposures and ageing processes. This holistic approach emphasises the following:
  ○ Prevention strategies;
  ○ Environmental policies;
  ○ Personalised interventions.

These strategies aim to mitigate the impact of environmental drivers on ageing and improve long-term health outcomes.

## 9. Exposome and the Secrets of Longevity: What We Can Learn from Blue Zones and Italian Longevity Hotspots

The concept of healthy ageing is exemplified by longevity hotspots known as Blue Zones. A Blue Zone (BZ) is a geographically small and relatively homogeneous area where people share a common lifestyle, environment, and likely a similar gene pool; their scientifically validated longevity surpasses that of surrounding regions. These areas are typically characterised by low pollution levels, a traditional way of life with minimal stress, strong family and community support, a natural diet poor in processed foods, and high physical activity levels even in old age. The inhabitants of each area, including Okinawa (Japan), Sardinia (Italy), Nicoya (Costa Rica), Ikaria (Greece), and Martinique (a France overseas region), share common genetic, environmental, dietary, and lifestyle factors that promote longevity [12,144]. Similarly, Cilento [11,145,146,147], a region in Southern Italy, and mountain villages (Sicani and Madonie Mountains) of Sicily [10,148,149,150] (Pes and Caruso, unpublished observations) have been identified as an emerging longevity model, where residents exhibit exceptionally low rates of CVDs, cognitive decline, and metabolic disorders, despite having an ageing population [10,151,152]. Understanding the role of the exposome in these regions provides crucial insights into how environmental and behavioural factors can mitigate the risks associated with NCDs.

### 9.1. Diet, Microbiota, and Metabolic Health

One of the most striking features of both the BZs and the Italian longevity hotspots is their adherence to traditional, minimally processed diets. These diets are rich in whole grains, legumes, nuts, and fresh vegetables, with low consumption of red meat and refined sugars. Extra virgin olive oil, a staple in Mediterranean regions, provides potent anti-inflammatory and antioxidant effects, offering protection against CVD, T2D, and cancer [10,11,124,149,153,154]. Additionally, this nutrient-rich diet fosters a diverse gut microbiota, which plays a crucial role in immune function, metabolic regulation, and neuroprotection, since other studies have shown that oldest individuals exhibit a higher abundance of beneficial gut bacteria, which help reduce systemic inflammation, improve glucose metabolism, and enhance resilience against age-related diseases [145,155].

### 9.2. Epigenetics and Longevity Pathways

While research on epigenetics in BZs is still limited, existing studies, in populations with similar lifestyle and dietary patterns, suggest that longevity in these regions may be influenced by epigenetic modifications linked to diet, physical activity, and environmental exposures [156].

### 9.3. Environmental Purity and Reduced Toxic Exposures

Unlike urbanised environments where air pollution, industrial chemicals, and processed foods dominate, the natural landscapes of longevity hotspots provide clean air, pure water sources, and reduced exposure to environmental toxicants.

- Lower air pollution reduces oxidative stress and prevents accelerated vascular ageing [157].
- Minimal pesticide use and organic farming enhance the nutrient density of locally sourced foods [158].
- Low exposure to EDCs, such as phthalates and bisphenol A (BPA), supports hormonal balance, reducing the risk of T2D, obesity, and reproductive disorders [132].

These factors contribute to lower overall toxic burden, delaying the onset of age-related diseases and supporting long-term health in both Cilento and the mountain villages of Sicily.

### 9.4. Prioritising Natural Rhythms, Sleep Quality, and Stress Reduction

Residents of these regions prioritise natural rhythms, aligning their daily routines with environmental cues that support healthy ageing [12,159]. Key lifestyle factors that contribute to this include the following:

- Consistent exposure to natural light, which regulates melatonin production and enhances sleep quality [160].
- Daily naps (siestas), particularly in Mediterranean regions, which are associated with reduced cardiovascular stress and improved cognitive function [11,161].
- Minimal artificial light exposure at night, helping to maintain sleep integrity and metabolic balance [162].

Furthermore, low chronic stress levels are a hallmark of these communities, attributed to a slower pace of life, strong social bonds, and deep cultural traditions [11,145,163]. Chronic stress is a driver of inflamm-ageing, and its reduction significantly lowers the risk of hypertension, Alzheimer’s disease (AD), and immune dysfunction [27,164].

### 9.5. Social Cohesion, Purpose, and Cognitive Longevity

One of the most underrated yet critical factors in longevity is social connection. The BZs and the Italian longevity hotspots share a strong sense of community, multigenerational cohabitation, and high levels of social engagement, all of which are linked to the following [10,11,12,145,148,149,150,163]:

- Lower depression rates and better mental health outcomes.
- A sense of purpose (“Ikigai” in Japan, “Plan de Vida” in Costa Rica, and “La Bella Vita” in Cilento), which has been shown to increase lifespan by reducing stress-related inflammation.
- Cognitive resilience, with lower rates of dementia and AD compared to global averages.

These findings underscore the psychosocial dimension of the exposome, demonstrating that well-being and longevity are deeply interconnected with environmental and lifestyle factors in these regions (Table 2).

#### The Exposome as a Blueprint for Healthy Ageing

As discussed below, the BZs and the Italian longevity hotspots serve as natural laboratories of longevity, offering compelling evidence that modifying environmental exposures, diet, stress levels, and lifestyle habits can profoundly influence the ageing process. While genetic predisposition plays a role, it is ultimately the exposome—encompassing diet, pollution exposure, microbiota, and social environment—that dictates health trajectories.

## 10. Conclusions: Implications for Preventive Medicine

The exposome, which encompasses environmental, occupational, and lifestyle factors, plays a crucial role in health and ageing. Key stressors such as air pollution, climate change, water contamination, microplastics and xenobiotic exposure contribute to immune dysregulation, chronic inflammation, and age-related diseases. Understanding their impact is essential for preventive medicine, as environmental influences, diet, lifestyle, and social determinants collectively shape long-term health [10,11,12,145,146,148,149,150,163,165,166,167].

A comprehensive, exposome-based prevention strategy is needed, with policies aimed at reducing air pollution, mitigating climate change, and limiting exposure to EDCs, to lower the risk of NCDs like CVDs, cancer, and metabolic disorders. From a geroscience perspective, which examines ageing biological mechanisms as drivers of chronic disease, modifying exposome factors offers a promising path to delaying age-related decline. Early-life interventions are particularly impactful, as epigenetic and metabolic programming are highly sensitive to environmental exposures. Research highlights biological ageing as the primary risk factor for most chronic diseases, making it critical to address environmental and behavioural accelerators such as oxidative stress, chronic inflammation, and metabolic dysfunction. Promoting a Mediterranean-style diet, regular physical activity, and the preservation of natural environments supports healthy ageing [10,11,145,146,148,149,150,168,169]. Physical activity, in particular, enhances quality of life in older adults by improving metabolic health, reducing inflammation, and fostering social engagement [10,12,145]. Additionally, personalised medicine, integrating exposomic data with genetic predispositions, can refine risk assessment and enable targeted prevention strategies [170,171].

The longevity models of Blue Zones, Cilento, and mountain villages in Sicily provide valuable insights into healthy ageing. These populations thrive on diets rich in polyphenols and antioxidants, active lifestyles, strong intergenerational social networks, and minimal exposure to environmental toxins and stress. Their way of life serves as a blueprint for public health strategies aimed at enhancing resilience against chronic diseases. Translating these insights into effective public health and preventive medicine initiatives is crucial for extending healthspan and delaying age-related diseases. Targeted interventions, such as pollution reduction, dietary improvements, and community-based health programmes, can promote healthier ageing on a global scale. Initiatives inspired by their lifestyle, including urban green spaces, clean food systems, and social engagement programmes, could significantly improve overall population health. Minimising exposure to air pollutants, endocrine disruptors, and ultra-processed foods, while promoting anti-inflammatory diets, regular physical activity, and stress management, could substantially lower age-related health risks. As urbanisation and modernisation introduce new challenges, preserving traditional lifestyle elements that support healthy ageing becomes increasingly important. Policies promoting local food production, sustainable agriculture, and pollution control may play a key role in maintaining these longevity benefits. Additionally, exploring how these longevity models can be adapted to diverse populations may offer valuable insights for global health strategies. The exceptional longevity observed in these communities suggests that a personalised approach, integrating environmental and cultural factors, may be more effective than generalised prevention strategies.

In conclusion, integrating exposome-based insights and geroscience principles into preventive medicine and public health is essential for reducing chronic disease burdens and promoting healthier ageing. This requires a holistic approach that combines environmental policies, public health strategies, and lifestyle modifications. Future research should explore how the exposome influences ageing at the molecular level, including its impact on gut microbiota, epigenetics, and metabolism. These insights should guide new research directions and inform targeted interventions. Moving forward, multisectoral policies that support environmental protection, lifestyle changes, and early-life interventions will be key to translating longevity science into longer, healthier lives for future generations.

## Figures and Tables

**Table 1:** Environmental challenges: health impacts and mitigation strategies.

Environmental Exposure	Health Impact	Potential Mitigation Strategies
Air Pollution (Particulate matter, NOx, SOx)	Chronic exposure linked to NCDs, cognitive decline, frailty, and mortality, especially in older adults.	Transition to renewable energy sources (solar, wind, geothermal).
	Increased all-cause mortality at PM2.5 levels below regulatory thresholds.	Implement stricter air quality standards and expand air monitoring networks.
	Vulnerability heightened by socioeconomic factors such as poverty, smoking, and obesity.	Address social determinants of health alongside pollution mitigation.
	Enhanced oxidative stress and systemic inflammation (inflamm-ageing).	Promote dietary antioxidants (e.g., sulforaphane, curcumin) to mitigate oxidative damage.
	Impaired cardiovascular health, including endothelial dysfunction and atherosclerosis [32,35,37,38].	Enforce vehicle emission regulations and encourage zero-emissions technologies.
Water Pollution (Heavy Metals, DBPs)	Toxicants like arsenic, lead, and cadmium inducing oxidative stress and mitochondrial dysfunction.	Improve water treatment infrastructure and remove persistent contaminants.
	Disruption of antioxidant pathways (e.g., Nrf2), leading to vascular inflammation and senescence.	Increase public awareness of water contaminants and encourage the use of water filtration systems.
	DBPs (e.g., THMs and HAAs) compounding oxidative stress and increasing cardiovascular risks [38,39,40,41,42,43,44,45,46,47,48,49,50,51,52,53,54,55,56,61,62,63,64,65,66,67,68].	Invest in cleaner water disinfection technologies to reduce by-product formation.
Microplastics	Inhalation and ingestion contribute to oxidative stress, inflammation, and cytotoxicity.	Regulate plastic waste and develop advanced filtration technologies.
	Can penetrate biological barriers, accumulating in vital organs and exacerbating systemic inflammation.	Improve monitoring of microplastic contamination in air and water.
	Linked to respiratory diseases such as lung cancer, asthma, and hypersensitivity pneumonitis.	Conduct further research on long-term health effects of microplastic exposure.
	Disrupts redox homeostasis by interfering with the Nrf2 pathway, increasing susceptibility to oxidative damage.	Encourage policies that limit microplastic release from industrial sources, synthetic textiles, and consumer products.
	Amplify the impact of other pollutants, thereby compounding health risks [70,71,72].	Develop mitigation strategies to counteract oxidative damage, such as antioxidant-based interventions.
Xenobiotics (Industrial Chemicals, Pharmaceuticals)	Endocrine disruption causing hormonal imbalances, developmental abnormalities, and increased cancer risk.	Strengthen regulations on industrial waste and pharmaceutical disposal, while restricting harmful industrial chemicals and promoting safer alternatives.
	Reduced detoxification capacity in ageing populations, increasing xenobiotic burden [73,74,75].	Promote genetic screening for vulnerabilities in xenobiotic-metabolising enzymes (XMEs).
	Epigenetic alterations (e.g., DNA methylation, miRNA dysregulation) exacerbating pollutant and heat-induced cellular damage [80,81,93].	Encourage research into epigenetic therapies and personalised medicine approaches.
Climate Change	Heat stress causing cardiovascular strain, oxidative stress, systemic inflammation.	Adaptive strategies (cooling centres, green infrastructure).
	Increased dehydration risk in older adults due to reduced thirst response and impaired renal function.	Promote hydration and implement public health campaigns.
	Amplified heatwaves due to heat domes, intensifying health risks for vulnerable populations.	Urban planning to mitigate heat island effects, such as increased vegetation and reflective building materials.
	Polonged exposure to high temperatures accelerates biological ageing, increasing the risk of disease and early mortality.	Implement cooling measures for vulnerable populations and strengthen monitoring of heat-related health effects.
	Crop yield reduction leading to malnutrition and micronutrient deficiencies. Climate change also facilitates the spread of infectious diseases by altering ecosystems and expanding the habitats of disease-carrying vectors [84,86,90,91,92,93,94,95,96,97,100,101].	Transition to climate-resilient agriculture and improve food security programmes.

Notes: PM2.5, PM10: particulate matter with diameter of 2.5 μm or less (PM2.5) and 10 μm or less (PM10); Nrf2: nuclear factor erythroid 2-related factor 2; ROS: reactive oxygen species; DBPs: disinfection by-products; XME: xenobiotic-metabolising enzyme; PAHs: polycyclic aromatic hydrocarbons. Health impacts of pollution and climate change are influenced by individual susceptibility, including age, genetics, and pre-existing health conditions.

**Table 2:** The science of longevity: BZs and Italian longevity hotspots as a model of healthy ageing.

Factor	Blue Zones	Cilento	Mountain Villages of Sicily	Biological Mechanisms and Health Benefits
Diet	Mediterranean-style diet rich in polyphenols, legumes, nuts, and olive oil (only in Ikaria and Sardinia); low in red meat and processed foods [11,145,165,166].	Traditional Mediterranean diet, rich in extra virgin olive oil, vegetables, whole grains; low in red meat and refined sugars [11,145,165].	Mediterranean diet with rural traditions, high consumption of legumes, wild herbs, seasonal vegetables, extra virgin olive oil; low in processed foods [10,148,150,151,153,165].	Antioxidant and anti-inflammatory effects, protects against CVD, T2D, neurodegenerative disorders [154,165].
Environmental Quality	Low pollution, clean air, pure water sources [12,147].	Low pollution, minimal industrial exposure, high environmental purity [145,146,147].	Low pollution, mountains climate with clean air, and natural water sources [10,151].	Reduces oxidative stress, supports mitochondrial function, prevents vascular ageing [159].
Natural Rhythms and Sleep	Exposure to natural light, daily naps (siestas), low artificial light at night [12,159].	Strong alignment with natural rhythmsand a culture of frequent napping [11,145].	Balanced natural rhythms due to rural lifestyle, frequent afternoon rest, low exposure to artificial light [10].	Improves sleep quality, lowers cortisol, enhances cognitive function [12,145,160].
Physical Activity	Daily natural movement (walking, farming, household activities) [11,12,145,166].	Active lifestyle with regular walking, gardening, and outdoor activities [11,145].	High physical activity through farming, walking, walking on hilly terrain, manual labour, and traditional crafts [10].	Reduces obesity risk, maintains cardiovascular health, enhances mitochondrial biogenesis [10,11,12,166].
Social Cohesion and Purpose	Strong community ties, family-centred lifestyle, “Ikigai” (sense of purpose), Plan de Vida [11,12,166,167].	Multigenerational living, strong social bonds, “La Bella Vita” philosophy [11,145,163].	Strong community ties, high family involvement, spiritual traditions, and social cohesion in small villages [10].	Lowers stress, improves mental resilience, enhances longevity [12,13,145,167].
Stress and Inflammation	Low chronic stress due to relaxed lifestyle and strong social support [11,12].	Low stress, strong community engagement [11,145].	Low stress due to rural environment, close-knit communities, and traditional spiritual practices [10].	Decreases systemic inflammation, lowers risk of age-related diseases [27,164].
Age-Related Diseases	Low incidence of CVD, T2D, and AD [11,12].	Low incidence of CVD, cognitive decline, and metabolic disorders [11,145,154].	Lower prevalence of CVD, metabolic syndrome, and cognitive disorders due to taditional diet and lifestyle [149,154].	Prolonged healthspan, lower morbidity, better quality of life [12,159].

## Data Availability

PubMed/Medline, Scopus, and Google Scholar.

## Abbreviations

The following abbreviations are used in this manuscript:

NCDs	Non-communicable diseases
CVDs	Cardiovascular diseases
PM2.5 and PM10	Particulate matter with diameter of 2.5 μm or less (PM2.5) and 10 μm or less (PM10)
NOx	Nitrogen oxides
Sox	Sulphur oxides
Nrf2	Nuclear factor erythroid 2-related factor 2
DNA	Deoxyribonucleic acid
DBPs	Disinfection by-products
THMs	Trihalomethanes
HAAs	Haloacetic acids
miRNA	Micro ribonucleic acid
T2D	Type 2 diabetes mellitus
WHO	World Health Organization
COPD	Chronic obstructive pulmonary disease
NF-κB24	Nuclear factor kappa B
AMPs	Antimicrobial peptides
LNs	Lymph nodes
EDCs	Endocrine-disrupting chemicals
BPA	Bisphenol and phthalates A
BZ	Blue Zone
ROS	Reactive oxygen species
XME	Xenobiotic-metabolising enzymes
PAHs	Polycyclic aromatic hydrocarbons
AD	Alzheimer’s disease
